# Mesenchymal Bone Marrow Cell Therapy in a Mouse Model of Chagas Disease. Where Do the Cells Go?

**DOI:** 10.1371/journal.pntd.0001971

**Published:** 2012-12-13

**Authors:** Jasmin, Linda A. Jelicks, Wade Koba, Herbert B. Tanowitz, Rosalia Mendez-Otero, Antonio C. Campos de Carvalho, David C. Spray

**Affiliations:** 1 Instituto de Biofísica Carlos Chagas Filho, Universidade Federal do Rio de Janeiro, Rio de Janeiro, Brazil; 2 Department of Neuroscience, Albert Einstein College of Medicine, Bronx, New York, United States of America; 3 Department of Physiology and Biophysics, Albert Einstein College of Medicine, Bronx, New York, United States of America; 4 Department of Radiology (Nuclear Medicine), Albert Einstein College of Medicine, Bronx, New York, United States of America; 5 Department of Pathology, Albert Einstein College of Medicine, Bronx, New York, United States of America; 6 Department of Medicine, Albert Einstein College of Medicine, Bronx, New York, United States of America; National Institutes of Health, United States of America

## Abstract

**Background:**

Chagas disease, resulting from infection with the parasite *Trypanosoma cruzi* (*T. cruzi*), is a major cause of cardiomyopathy in Latin America. Drug therapy for acute and chronic disease is limited. Stem cell therapy with bone marrow mesenchymal cells (MSCs) has emerged as a novel therapeutic option for cell death-related heart diseases, but efficacy of MSC has not been tested in Chagas disease.

**Methods and Results:**

We now report the use of cell-tracking strategies with nanoparticle labeled MSC to investigate migration of transplanted MSC in a murine model of Chagas disease, and correlate MSC biodistribution with glucose metabolism and morphology of heart in chagasic mice by small animal positron emission tomography (microPET). Mice were infected intraperitoneally with trypomastigotes of the Brazil strain of *T. cruzi* and treated by tail vein injection with MSC one month after infection. MSCs were labeled with near infrared fluorescent nanoparticles and tracked by an *in vivo* imaging system (IVIS). Our IVIS results two days after transplant revealed that a small, but significant, number of cells migrated to chagasic hearts when compared with control animals, whereas the vast majority of labeled MSC migrated to liver, lungs and spleen. Additionally, the microPET technique demonstrated that therapy with MSC reduced right ventricular dilation, a phenotype of the chagasic mouse model.

**Conclusions:**

We conclude that the beneficial effects of MSC therapy in chagasic mice arise from an indirect action of the cells in the heart rather than a direct action due to incorporation of large numbers of transplanted MSC into working myocardium.

## Author Summary

Chagas disease, resulting from infection with the parasite *Trypanosoma cruzi*, is a major cause of heart disease in Latin America. Treatment options are limited to a small number of drugs that were developed more than four decades ago and which have various drawbacks. Stem cell therapy with bone marrow mesenchymal cells (MSCs) has emerged as a novel therapeutic option for cell death-related heart diseases, but efficacy of MSCs has not been tested in Chagas disease therapy. Due to the established role of the immune system in the physiopathology of Chagas disease and the immune modulatory properties of MSC we hypothesized that MSC could be an optimal cell type for therapy in chagasic cardiomyopathy. Therefore, in this study we have used cell tracking strategies following labeling of MSCs with nanoparticles to investigate migration of transplanted MSCs in a murine model of Chagas disease, and have correlated MSCs migration with cardiac function in chagasic animals by small animal positron emission tomography imaging technique.

## Introduction

Chagas disease is a serious public health problem in all Latin American countries [1], where it is estimated that 15–16 million people are infected with the its causative agent, the parasite *Trypanosoma cruzi* (*T. cruzi*) [2]. Although *T. cruzi* is endemic in Latin America, thousands of people are infected in Europe, United States, Canada, among other countries, due to migration of infected people [3], [4].

Approximately one-third of individuals with Chagas disease develop a symptomatic chronic phase decades after the infection, of which 90% develop heart disease and the other 10% are affected by gastrointestinal diseases [5]. Chronic Chagas heart disease is a progressive, fibrotic inflammatory cardiomyopathy that results in permanent heart damage [6]. This heart damage leads to dilation and cardiac arrhythmia, and ultimately to congestive heart failure, which is the primary cause of death in chronic Chagas heart disease patients [7], [8]. For more than 40 years, the only treatment option for Chagas disease in the acute phase has been the anti-parasitic drugs nifurtimox and benznidazole. However, these drugs have side effects and lead to parasite resistance [9]. In the chronic phase, when congestive heart failure ensues, heart transplantation is often the only therapeutic option, which is also fraught with many problems.

In this complex scenario, where an estimated 20,000 people die of chronic Chagas heart disease each year [1], cell therapies appear as an alternative solution. In a mouse model of chronic chagasic cardiomyopathy (CCC) we have previously shown that mononuclear cells from the bone marrow decrease inflammation and fibrosis, reduce or reverse right ventricular dilation and significantly restore gene expression pattern to that of control, non-infected hearts [10]–[12]. However, given the established role of the immune system in the physiopathology of Chagas disease [13] and the immune modulatory properties of bone marrow mesenchymal cells (MSC) [14] we hypothesized that MSC could be an optimal cell type for therapy in chagasic cardiomyopathy. In addition, preliminary studies with mononuclear cells from chronic chagasic patients have revealed a diminished colony forming capacity (unpublished data), which can compromise autologous therapy. Due to the immune privileged characteristics of MSC, these cells can be used as an allogenic product [15]. Furthermore, previous studies with cellular therapy have focused primarily on the chronic phase of the disease and data about the effect of cellular therapy at early stages, such as 1 month after infection, was not previously evaluated. Thus, we wanted to examine the hypothesis that cell therapy is effective at earlier stage of the disease.

Therefore, in this study we describe the use of cell tracking strategies following labeling of MSC with nanoparticles to investigate migration of intravenously transplanted cells in an acute murine model of *T. cruz*i infection. Furthermore, we correlated MSC migration with glucose metabolism and morphology of heart in chagasic mice by small animal positron emission tomography (microPET)**.**

## Materials and Methods

### Animals

All experiments were performed on adult male CD-1 mice in accordance with the U.S. National Institutes of Health Guide for the Care and Use of Laboratory Animals (NIH Publication No. 80-23), approved by the Institutional Animal Care and Use Committee of the Albert Einstein College of Medicine.

### Isolation and Cultivation of Mesenchymal Cells from Bone Marrow

To obtain bone marrow cells, tibias and femurs of approximately 8 week old mice were isolated, the epiphyses were removed, the bones were individually inserted in 1 mL automatic pipette polypropylene tips and then put in 15 mL tubes. The bones were centrifuged at 300× g for 1 min and the pellets suspended in Dulbecco's modified Eagle's high glucose medium (DMEM; Invitrogen Inc., Carlsbad,CA), supplemented with 10% fetal bovine serum (FBS; Invitrogen Inc.), 2 mM l-glutamine (Invitrogen Inc.), 100 U/mL penicillin (Sigma-Aldrich Co., St. Louis, MO), and 100 µg/mL streptomycin (Sigma-Aldrich). The cells were plated in 100 mm culture dishes with supplemented DMEM and maintained in 5% CO_2_ atmosphere at 37°C. The medium was replaced 48–72 hrs after initial culture to remove non adherent cells and the adherent cells were grown to confluence before each passage. Medium was replaced three times a week. All experiments were performed on second or third passage cells.

### Mesenchymal Cell Labeling

In the present study we used fluorescent nanoparticles called X-Sight nanospheres (Carestream Health Inc., Rochester, NY): X-Sight 761 (761 nm excitation and 789 nm emission) and X-Sight 549 (549 nm excitation and 569 nm emission). We incubated MSC with a solution of 0.3 mg/mL X-Sight in supplemented DMEM in 5% CO_2_ atmosphere at 37°C for 4 hours.

The labeled cells with X-Sight were then washed three times with phosphate-buffered saline (PBS), trypsinized and centrifuged at 300× g for 5 min. Subsequently, the labeled cells were used for *in vitro* experiments or for tracking after transplant.

### *Trypanosoma cruzi* Infection and Cell Therapy

The Brazil strain of *T. cruzi* was maintained by serial passage in C3H mice (Jackson Laboratories, Bar harbor, ME). Eight to 10 week old male CD-1 mice (Charles River) were infected by intraperitoneal injection of 5×10^4^ trypomastigotes in saline solution. One month after infection (1MAI) these mice received a single dose of 3×10^6^ MSC in 100 µL of PBS, or 100 µL of PBS via tail vein. For cell tracking, both control and chagasic mice received single doses of 3×10^6^ labeled MSC via tail vein.

### Cell Visualization by *In Vivo* Imaging System

The X-Sight 761-labeled MSC were visualized by the *in vivo* imaging system (IVIS) Kodak Image Station 4000MM PRO (Carestream Health) equipped with a CCD camera. For the fluorescence imaging, the machine was configured for 760 nm excitation, 830 nm emission, 3 min exposure, 2×2 binning and f-stop 2.5. The acquired images were analyzed with the Carestream MI Application 5.0.2.30 software (Carestream Health).

#### *In vitro* imaging

We performed *in vitro* imaging of X-Sight 761-labeled cells to determine the minimal number of cells that can be visualized by the IVIS technique and the retention time of the particles. For this propose, the MSC were incubated with X-Sight 761 in a 100 mm culture dish, trypsinized and plated in 96-well plate at multiple concentrations. The analyzed concentrations were 5×10^3^, 10^4^, 5×10^4^, 10^5^ and 5×10^5^ cells/well and the images were acquired 2 hours, 2 days and weekly up to 4 weeks after plating in the 96-well culture plate.

#### Tracking X-Sight 761-labeled mesenchymal cells

Whole body images were acquired from the ventral surface of the mice. Due to prior knowledge that the IVIS technique has limited penetration depth and poor spatial resolution, we subsequently isolated organs of interested, including heart, bladder, lung, liver, spleen and kidney to perform *ex vivo* imaging. The images were acquired 2 or 15 days after labeled cell (MSC761 2d or MSC761 15d) transplantation in control or infected mice one month post infection. Eight mice were used in the control group and 4 mice were used in the other groups.

### Cell Visualization by Confocal Microscopy

For *in vitro* visualization of labeled cells, the MSC were grown on glass coverslips coated with 0.2% gelatin, incubated with X-Sight 549 for 4 hours, washed with PBS and fixed for 20 min in 4% paraformaldehyde. The cells were then observed by confocal microscopy to ascertain intracellular incorporation of the particles.

Besides the IVIS technique, we tracked the labeled cells in the heart by microscopy. The same hearts used for IVIS tracking were fixed overnight in 4% paraformaldehyde, incubated in optical cutting temperature resin (Sakura Finetek USA, Inc., Torrance, CA) and sliced in 5 µm frozen sections. The photomicrographs shown in this study were obtained using a Zeiss LSM 510 Duo confocal microscope.

### Positron Emission Tomography

Mice were administered 300–400 µCi (12–15 MBq) of [^18^F] fluoro-2-deoxyglucose (^18^F -FDG) in 100 µL saline via tail vein and imaging was started 1 hour after injection. Imaging was performed on an Inveon Multimodality scanner (Siemens Healthcare, Erlagen, Germany) using its PET module. The mice were anesthetized with isoflurane inhalation anesthesia (2% in 100% oxygen) administered via a nose cone. PET imaging was performed using the PET gantry which provides 12.7 cm axial and 10 cm transaxial active field of view. The PET scanner has no septa and acquisitions are performed in 3-D list mode. A reconstructed full-width-half-maximum (FWHM) resolution of <1.4 mm is achievable in the center of the axial field of view. After each acquisition (approximately 3 minutes), data were sorted into 3D sinograms, and images were reconstructed using a 2D-Ordered Subset Expectation Maximization algorithm. Data were corrected for dead-time counting losses, scatter, random coincidences and the measured non-uniformity of detector response (i.e., normalized) but not for attenuation. Analysis was performed using ASIPRO and IRW (Siemens Healthcare) dedicated software.

The images were acquired from animals at 1 month after infection (before treatment) and 15 and 30 days after treatment with PBS or cells. Control group imaging was performed at every time point and for statistical analysis they were combined, thus, the technical number of control animals was 12. In addition, as the collected data at 15 days after treatment was very similar to the collected data at 30 days, we combined these time points to increase the sample number in a group called 15–30d, thus, the technical number of animals were 8 and 9 for the PBS and MSC treated groups, respectively.

### Statistical Analysis

Statistical significance was evaluated using one-way ANOVA with Newman-Keuls post-test for comparison among multiple groups. All calculations were done using GraphPad Prism 5 for Windows (GraphPad Software, San Diego, CA) and p<0.05 was considered as statistically significant. The data are presented as mean and the error bars represent the standard error of the mean.

## Results

### Efficient Labeling of Mesenchymal Cells *In Vitro*

**Figure 1:**
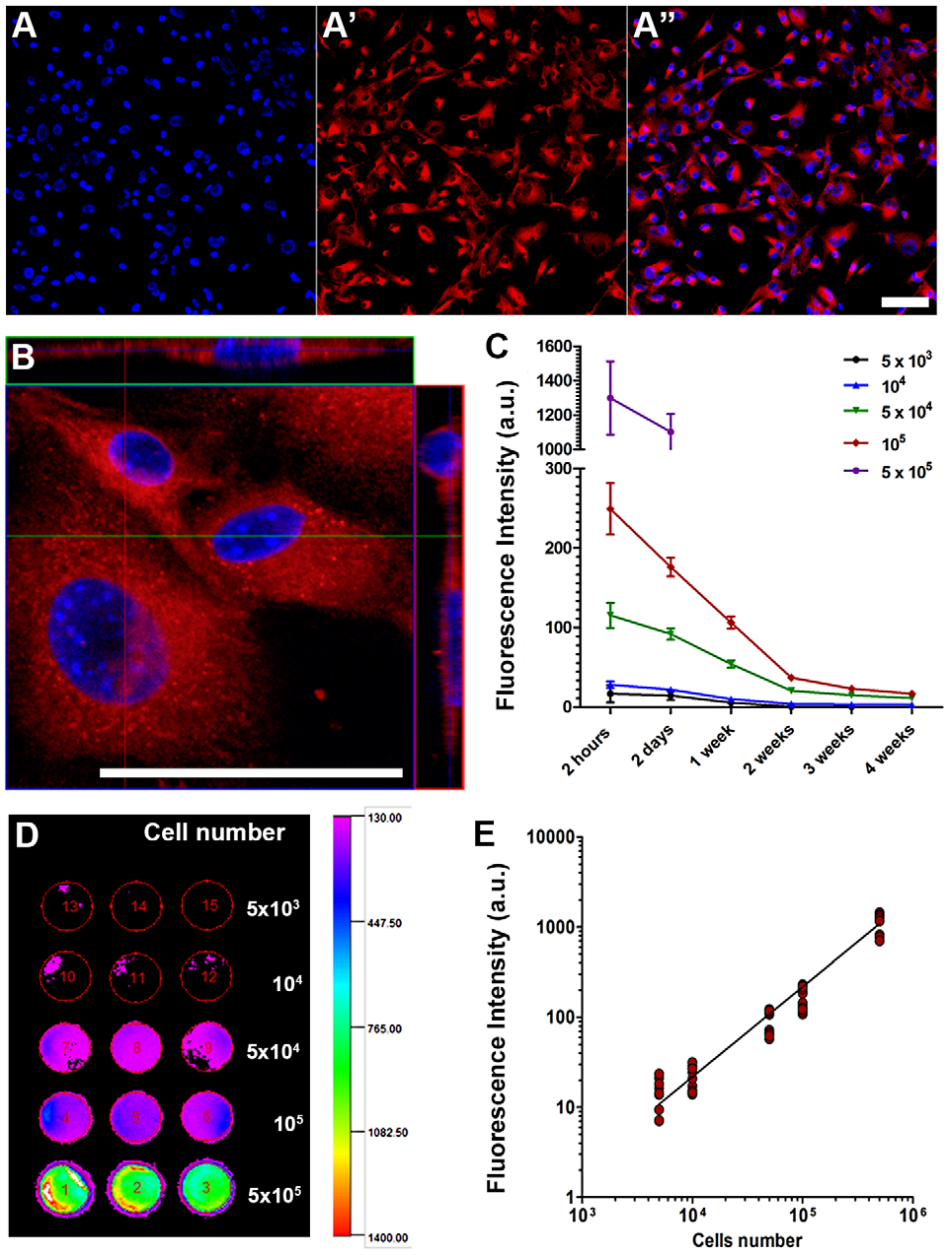
Detection of X-Sight761 nanoparticles into MSCs *in vitro*. X-Sight incorporation was observed by IVIS and confocal microscopy. (**A-A″**) Fluorescence microscopy images showing the high efficiency of X-Sight to label MSCs after 4 hours of incubation. (**A**) Staining wih the nuclear stain DAPI. (**A′**) X-Sight nanoparticles. (**A″**) Merge of images showing that apparently all MSCs incorporated nanoparticles. (**B**) Confocal microscope image showing, at high magnification, that X-Sight nanoparticles were incorporated into cell cytoplasm. (**C–E**) Detection of labeled MSCs by IVIS technique in 96-well plate. (**C**) Analysis of retention time of X-Sight for up to 4 weekes using different cell plating densities. (**D**) Representative IVIS image showing labeled cells at different concentrations in a 96-well plate, 2 days after intial exposure of 4 hours. (**E**) Graph showing a linear correlation between the number of cells and fluorescence mean intensity of the same cells represented in image (D). Scale bar in image (A″) and (B) = 50 µm.

By microscopy, we observed that all of the cells were labeled with X-Sight nanoparticles *in vitro* after 4 hours of incubation (Figure 1A-A″) and because of that we considered that it was not necessary to quantify the number of labeled cells. By confocal microscopy, we confirmed that the nanoparticles were incorporated into the cell cytoplasm (Figure 1B). We did not observe a cytotoxic effect of the X-Sight on cellular proliferation, evaluated by ki67 antibody, or on viability, evaluated by trypan blue staining (data not shown). We analyzed the retention time of nanoparticles *in vitro* for up to 4 weeks using different cell plating densities, and we observed a substantial loss of fluorescence intensity over time (Figure 1C), likely due to cellular proliferation as previously described by us [16]. The wells plated at 5×10^5^ cells could not be monitored beyond 2 days because of high confluence and, consequently, cellular death. However lower plating densities allowed us to detect signals for up to 1–4 weeks. At 2 days after initial exposure to X-Sight for 4 hours a direct relation between cell number and fluorescence intensity was observed and a small number of cells, as low as 5×10^3^ was detected (Figure 1D and E).

### Tracking X-Sight 761-Labeled Cells

**Figure 2:**
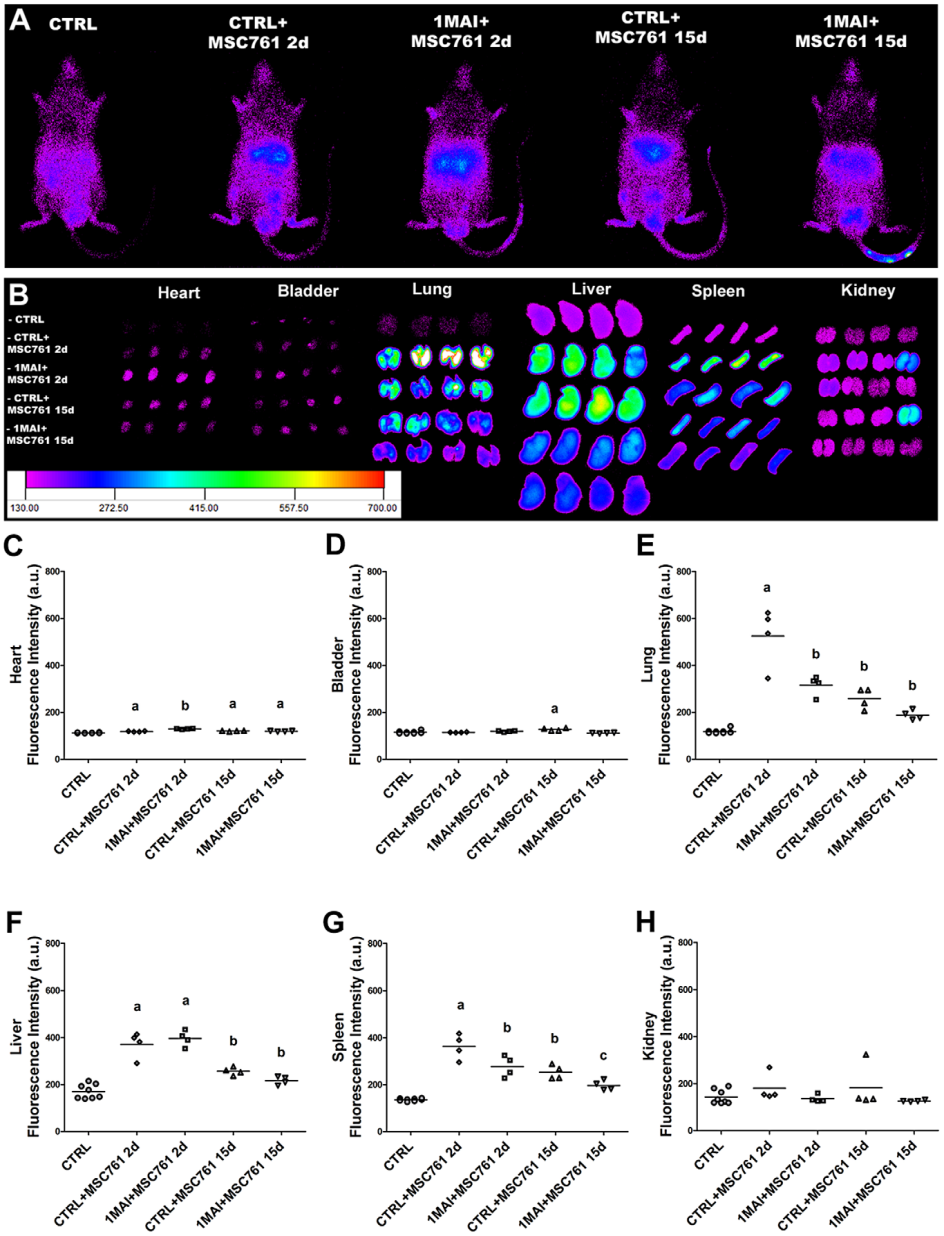
Tracking of X-Sight-labeled MSCs in control and chagasic mice 2 or 15 days after cell transplantation. The cells were intravenously injected (tail vein) in control and chagasic animals 1 month after infection for tracking by IVIS. (**A**) Representative images showing the distribution of transplanted MSCs viewed from in the ventral surface of the body. (**B**) Images of *ex vivo* organs showing the distribution of labeled MSCs. Note the majority of the signal is localized in the lung, liver and spleen of the animals. (**C–H**) Quantification of fluorescence intensity of *ex vivo* organs shown in images (B). The evaluated organs were (**C**) heart, (**D**) bladder, (**E**) lung, (**F**) liver, (**G**) spleen and (**H**) kidney. *P*<0.05.

Control and chagasic mice at 1 month post infection received X-Sight 761-labeled MSC via tail vein. The images were acquired by IVIS, 2 or 15 days after cell transplantation. A weak signal from the labeled cells was observed in whole body images (Figure 2A) and a better signal was detected in *ex vivo* organs (Figure 2B and H). Despite the filters being set to near infrared excitation and emission, a basal level of fluorescence was detected in control mice (Figure 2A-CTRL), which did not receive cells, and similar fluorescence intensity was found in the infected mice that received unlabeled cells, indicating that neither the disease nor the unlabeled cells affected the basal fluorescence (data not shown).

From all analyzed organs, including heart, bladder, lung, liver, spleen and kidney we observed that approximately 70% of the fluorescence was localized in the lung, liver and spleen of control and chagasic mice (Figure 2C–H). We also harvested some tissue samples, including leg muscle, white and brown adipose tissue but we did not observe cell migration to these tissues (data not shown). When we compared the fluorescence intensity in the organs, 2 and 15 days after transplantation, a decrease of approximately 60% in total intensity was seen (Figure 2C–H). Based on the possibility that nanoparticles might be released and secondarily label other cells, such as macrophages, we injected free X-Sight in the animals. In contrast to the distribution of labeled cells, free X-Sight was distributed more widely in whole body and about 60% of signal was found exclusively in the liver (data not shown).

**Figure 3:**
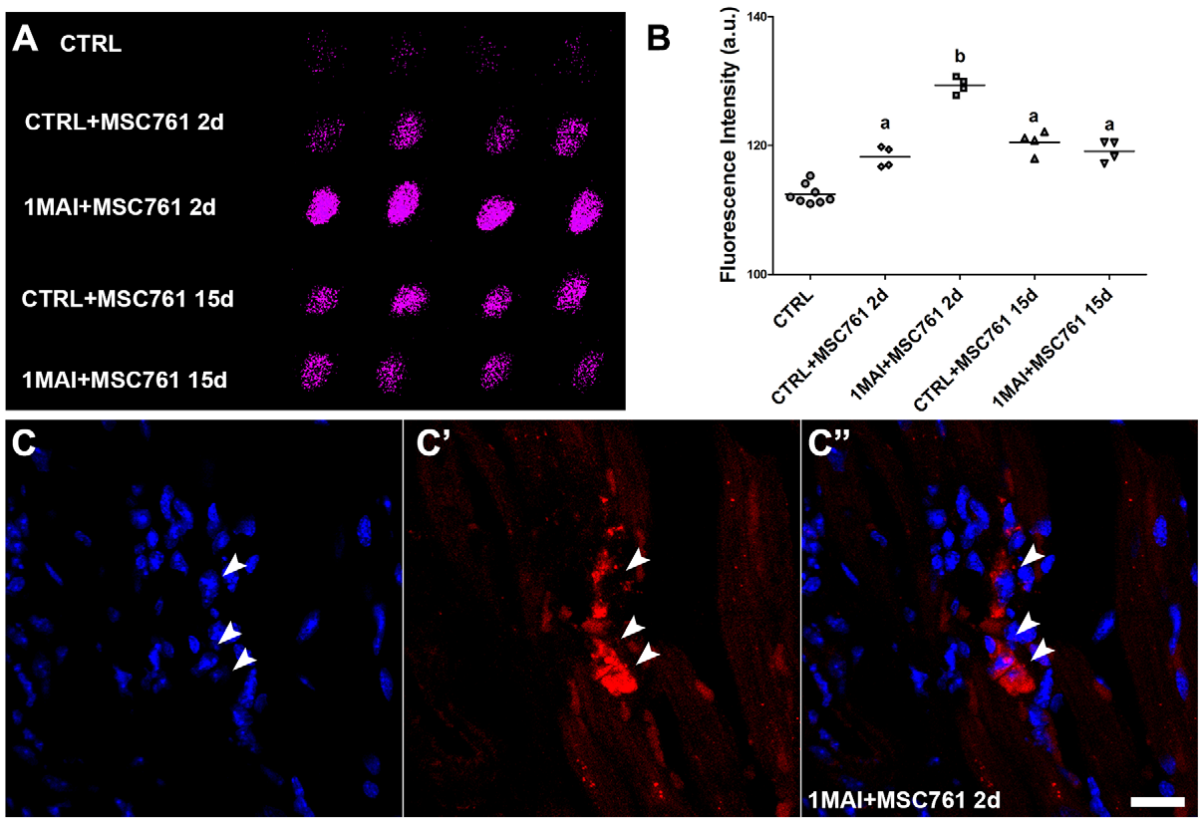
Tracking of labeled MSCs with X-Sight in the hearts of control and chagasic mice 2 or 15 days after cells transplantation. Although the majority of the signal is localized in the lung, liver and spleen of the animals it was possible to observe labeled cells in the heart. (**A**) Same *ex vivo* heart images shown in Figure 2B represented in high magnification showing a greater number of cells migrated to chagasic hearts when compared with control hearts. (**B**) Statistical analysis of heart fluorescent intensity represented in (A). We observed a stronger signal in the chagasic hearts 2 days after transplantation when compared to the others groups, suggesting that there is a preferential migration to the damage tissue. (**C-C″**) Representative confocal microscopy images showing X-Sight-labeled cells (arrows) in heart slice of chagasic animals. Only a small number of labeled cells was found in the slices. (**C**) DAPI. (**C′**) X-Sight nanoparticles. (**C″**) Merge of images. Scale bar = 20 µm. *P*<0.05.

It was interesting to note that despite the fluorescence signal in lung and spleen was stronger in control animals than in chagasic animal 2 days after therapy (Figure 2E and H), in the heart we noticed the opposite. The quantification of fluorescence intensity showed that signal from chagasic hearts was statistically higher when compared to hearts obtained from control mice when *ex vivo* images of heart were compared 2 days after transplantation (Figure 2C), suggesting the homing of cells to the, most affected tissue by the disease (Figure 2C). In Figure 3A and B it is possible to observe with more detail the *ex vivo* images and graph of the hearts evaluated. In histological examination of the heart there were rare X-Sight-labeled cells in this tissue by confocal microscopy (Figure 3C-C″), which corroborates our data shown in Figure 2B–H where it is possible to note that only few cells migrate to this organ when compared to lung, liver and spleen.

All analyzed organs in this experiment were weighed and wet weights were found not to be affected by the infection, except the spleen. The spleens of the chagasic animals were heavier than control, independent of PBS or MSC treatment for 1 month (84.35±2.5; 236.4±23.7 and 229.9±30.9 mg, for control or chagasic mice treated with PBS or MSC, respectively).

### Positron Emission Tomography

**Figure 4:**
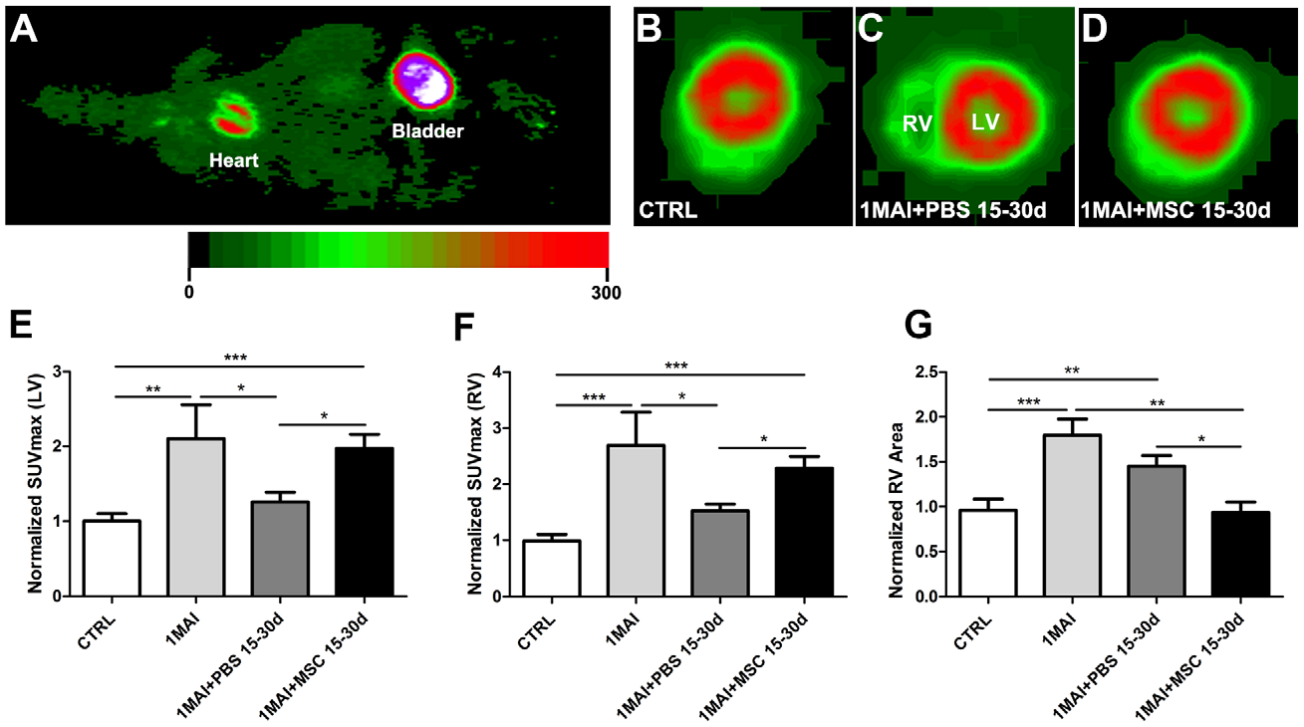
Analyses of metabolic and morphological myocardium state using microPET technique. Infected animals with *T. cruzi* for 1 month were treated with PBS or MSC and analyzed 15–30 days after transplantation. The cardiac assessment was obtained by regional uptake of the glucose analogue ^18^F-FDG. (**A**) Representative image of the whole body, horizontal plane, assessed by microPET technique of a control animal. Note the high glucose activity in the heart. (**B–D**) Transversal plane images of the heart in high magnification showing the right and left ventricles. It is possible to observe right ventricle dilation in the untreated chagasic animal in image (C). (**E–F**) Graphs showing the glucose uptake in (E) left and (F) right ventricles. (**G**) Graph demonstrating the dilation in the right ventricle of infected mice and the recovery by MSC therapy. *P<0.05, **P<0.01 and ***P<0.001.

MicroPET was performed with two main goals: to evaluate the glucose metabolism of the heart using the radioactive tracer ^18^F-FDG, and to measure the right ventricle (RV) dilation which is typical of the murine Chagas disease model [12], [17]. Figure 4A represents a whole body image, in a horizontal plane, from an animal that received ^18^F-FDG. In high magnification, in a transversal plane, it was possible to visualize the heart of control (Figure 4B), mice infected for 1 month and treated with PBS (Figure 4C) or MSC (Figure 4D). Note the size of the RV in Figure 4C, from an untreated mouse. A high glucose activity was observed 1 month after infection (without treatment) in the LV (Figure 4E) as well as in the RV (Figure 4F). However, 15–30 days after PBS treatment, a decrease in the glucose activity was observed in both ventricles what was not observed in the MSC treated groups. These data indicate that MSC can increase the glucose metabolism in infected hearts. Besides the heart, we observed a high glucose uptake in the brain, but we found no difference in uptake among the different experimental groups (data not shown). When the RV area was measured, the dilation observed in the RV of mice 1 month post infection was significantly reduced by cell therapy 15–30 days after cell transplantation (Figure 4G).

## Discussion

There are two types of cell therapy approaches that have been applied to animal models of Chagas disease: bone marrow mononuclear cells in mice [10]–[12] and co-cultured skeletal myoblasts with MSC in rats [18]. In human clinical studies of patients with end-stage heart failure due to Chagas disease the administration of autologous bone marrow mononuclear cells did not improve cardiac function [19]. The transplant of skeletal myoblast was associated with cardiac arrhythmias due to their inability to form electric coupling with cardiac myocytes [20]. Thus, these cells are not recommended for a disease with high incidence of arrhythmias, such as Chagas disease. On the other hand, MSC electrically couple to host cardiac myocytes and they have been suggested as a better cell type for cardiac therapy than other cell types, such as skeletal myoblasts [15].

MSC is an immune privileged cell type which can interact with cells of both the innate and adaptive immune systems and release trophic factors [14], [21]. Hence, MSC might modulate the inflammation and reverse the tissue damage caused by *T. cruzi* infection. However there is no previous report of therapy for Chagas disease using only MSC. The present study thus pioneers the analysis of MSC therapy and biodistribution of these cells in infected mice.

In contrast to myocardial infarction which causes a regional damage, Chagas disease affects the heart globally. Therefore, systemic delivery of MSC has been chosen for small animals infected with *T. cruzi* since multiple local injections into several heart areas would be expected to generate tissue damage [22]. Thus, it is very important to identify the preferential sites of MSC migration and correlate with their effects in cardiac function. In the present study an efficient visualization of X-Sight-labeled MSC was obtained and a small cell number as low as 5×10^3^ could be detected *in vitro* by IVIS. However, there was a rapid decrease in fluorescence intensity over time. Based on our previous study with MSC labeled with superparamagnetic oxide iron nanoparticles [16], cellular proliferation seems largely responsible for the signal decrease observed *in vitro*. Regarding cell homing to the site of infection, we observed that migration was significantly higher to the hearts of infected mice when compared to controls. MSC migration to the damaged tissue has been reported by several authors in different models such as tumors [23], arthritic joints [24], middle cerebral artery occlusion [25] and myocardial infarction [26]. Although MSCs reside in specialized niches their perivascular location allows global access to the tissues and when they migrate to an injured region they may secrete large amounts of immune regulatory and trophic bioactive factors [21]. The exact mechanisms and molecules involved in migration of MSCs to areas of inflammation are unknown. It is assumed that the process of MSC migration is similar to that of leukocytes [27]. This process comprises different types of molecules such as chemokines and their receptors, adhesion molecules and proteases [28]. The increase in chemokine concentrations at the site of inflammation is crucial for the MSC migration to injury site. The stromal cell-derived factor-1 (SDF-1) is a member of the inflammatory chemokine family and stimulates the migration of various progenitor cells to injury site, including hematopoietic stem cells and MSCs, due to the CXC chemokine receptor type 4 (CXCR4) [29].

Although some cells migrate to the heart, as visualized by IVIS and confocal microscopy, the quantity is negligible when compared to liver, lungs and spleen, where about 70% of the fluorescence intensity was found. Our findings are consistent with other studies where the majority of intravenous injected MSC were found in the liver, lung and spleen, including in dogs with myocardial infarction [26] and patients with cirrhosis [30]. The fluorescence intensity 15 days after transplantation was greatly reduced, which is likely due to nanoparticle exocytosis, cellular proliferation and/or death [31]. It has been shown that most MSC die within days or weeks of transplantation, yet their beneficial effects can be seen over a much longer term, suggesting a critical time window for MSC action [32].

The radioactive tracer ^18^F-FDG has been used to analyze the area of infarcted myocardium in mouse [33] and humans [34] and seems to be helpful in the diagnosis of infection and inflammation [35]. Here we used the ^18^F-FDG technique to evaluate glucose metabolism and morphology of the hearts by microPET. ^18^F-FDG uptake was increased in chagasic animals 15–30 days after infection and decreased to control levels 45–60 after infection (1MAI + PBS 15–30d group). Although these data differ from those of a previous study from our group, which showed the increase of ^18^F-FDG uptake in all time points studied [17], in both studies there is a peak of uptake at 15–30 days after infection that corresponds to the peak of parasitemia, 25–30 days after infection in this model [36]. Although the incorporation of ^18^F-FDG is related to the general glucose metabolism, several authors suggest that the incorporation increase in tissues may also be related to inflammation and infection events [35], [37]. It was interesting to note that MSC therapy increased ^18^F-FDG uptake in the heart, since the number of MSC present in this organ is very low, as revealed in the tracking experiment we suggest that the MSC induced increase in ^18^F-FDG uptake is due to the known effects of MSC in damaged tissues, such as enhanced angiogenesis, stimulation of mitosis in stem and progenitor cells, recruitment of circulating stem cells, inhibition of apoptosis and/or change in extracellular matrix composition [14], [38]. We performed western blot analyses of heart tissue which reveled that MSC did not modify inflammatory proteins, such as interferon-γ (INF- γ) and interleukin 1β (IL-1β) and 10 (IL-10) in chagasic animals at a time point 1 month after infection with 1 month of therapy (total of 2 months of infection). Evaluating another time point of treatment (at 2 months after infection) we did not observe alterations in these proteins at 1 month after treatment (total of 3 months of infection) either; however, we did note differences in INF- γ and IL-10 due to MSC therapy after 2 months of treatment (total of 4 months of disease). Thus, despite the fact that we did not observe an immunomodulation after 1 month of therapy, we have obtained evidence that MSCs are able to immunomodulate after a longer term (data not shown). The remodeling of the right ventricle (RV) has been shown to be a characteristic phenotype of the chagasic mouse model used by our group [12], [17], [36], [39]. RV dysfunction was described as a predictor of mortality in patients with chagasic cardiomyopathy [40]. Regarding the heart morphology evaluation by microPET, we observed that the RV dilation caused by *T. cruzi* infection was reduced after cellular therapy. This result demonstrates that MSC therapy is able to reduce the RV dilation in Chagas disease model and corroborates with another study from our group, in which magnetic nuclear resonance was used to show that RV dilation was reduced after bone marrow mononuclear cell transplantation [12].

To summarize, this study is the first to use MSC for therapy and cell tracking in chagasic mice. It was interesting to note that despite a very small number of cells migrating to the heart when compared to the other organs, a statistically significant preferential migration to the damage heart was observed. Since the vast majority of the intravenous injected cells migrated to lung, liver and spleen we suggest that the beneficial effect observed by MSC cell therapy in chagasic mice is due to an indirect action of the cells in the heart rather than a direct action by incorporation of large numbers of MSC into the working myocardium.
